# Integrated Functional Neuroimaging, Monoamine Neurotransmitters, and Behavioral Score on Depressive Tendency in Intensive Care Unit Medical Staffs Induced by Sleep Deprivation After Night Shift Work

**DOI:** 10.3389/fpsyt.2022.848709

**Published:** 2022-03-22

**Authors:** Haotian Ye, Muhuo Ji, Chaoyan Wang, Cong Wang, Ying Li, Yuan Chen, Lisha Cheng, Yanfei Li, Jian-Jun Yang

**Affiliations:** ^1^Department of Anesthesiology, Pain and Perioperative Medicine, The First Affiliated Hospital of Zhengzhou University, Zhengzhou, China; ^2^Department of Anesthesiology, The Second Affiliated Hospital, Nanjing Medical University, Nanjing, China; ^3^Department of Magnetic Resonance Imaging, The First Affiliated Hospital of Zhengzhou University, Zhengzhou, China; ^4^Department of Anesthesiology, Jiangyin Hospital, Affiliated to Southeast University Medical School, Jiangyin, China; ^5^Department of Neurology, The First Affiliated Hospital of Zhengzhou University, Zhengzhou, China

**Keywords:** shift work, sleep deprivation, resting-state fMRI, depression, functional connectivity density, monoamine neurotransmitters

## Abstract

**Background:**

Intensive care unit (ICU) medical staffs undergoing sleep deprivation with perennial night shift work were usually at high risk of depression. However, shift work on depression-related resting-state functional magnetic resonance imaging was still not fully understood. The objective of this study was to explore the effects of sleep deprivation in ICU medical staffs after one night of shift work on brain functional connectivity density (FCD) and Hamilton Depression Rating Scale (HAMD) scores. Also, serum neurotransmitter concentrations of serotonin (5-HT) and norepinephrine (NE) were obtained simultaneously.

**Methods:**

A total of 21 ICU medical staffs without psychiatric history were recruited. All participants received HAMD score assessment and resting-state functional magnetic resonance imaging scans at two time points: one at rested wakefulness and the other after sleep deprivation (SD) accompanied with one night of shift work. Global FCD, local FCD, and long-range FCD (lrFCD) were used to evaluate spontaneous brain activity in the whole brain. In the meantime, peripheral blood samples were collected for measurement of serum 5-HT and NE levels. All these data were acquired between 7:00 and 8:00 am to limit the influence of biological rhythms. The correlations between the FCD values and HAMD scores and serum levels of neurotransmitters were analyzed concurrently.

**Results:**

Functional connectivity density mapping manifested that global FCD was decreased in the right medial frontal gyrus and the anterior cingulate gyrus, whereas lrFCD was decreased mainly in the right medial frontal gyrus. Most of these brain areas with FCD differences were components of the default mode network and overlapped with the medial prefrontal cortex. The lrFCD in the medial frontal gyrus showed a negative correlation with HAMD scores after SD. Compared with rested wakefulness, serum levels of 5-HT and NE decreased significantly, whereas HAMD scores were higher after SD within subjects.

**Conclusions:**

Our study suggested that sleep deprivation after night shift work can induce depressive tendency in ICU medical staffs, which might be related to alterative medial prefrontal cortex, raised HAMD scores, and varying monoamine neurotransmitters.

## Introduction

Intensive care unit (ICU) physicians are typically perceived to be perfectionist and comprehensive professionals (1). Long working hours and high frequencies of monthly on-duties are commonly found in ICU medical staffs (2). Thus, sleep deprivation (SD) becomes an inherent problem to shift work, which usually leads to personal desynchronization of circadian rhythms (3). SD has a profound influence on gastrointestinal and cardiovascular diseases, diabetes, obesity, certain kinds of cancer, and neuropsychiatric disorders (4–9). Even acute short-term SD can impair vigilance, attention, executive functions, working memory, and cognitive abilities (10, 11). Heavy workload could be aggravated by night shift-related SD, which can decline their performance and impact on patient's safety (12). In one previous study, it was reported that the tracheal intubation time taken by physicians was significantly longer during a night shift than during the daytime (13). Also, SD can increase anxiety, irritability, and depression scores (14). One study conducted in French ICUs concluded that nearly a quarter of healthcare staff suffered from depression (15). In another study, approximately one-third of United Kingdom ICU physicians were subjected to depression (16). However, the neural mechanism underlying shift work-induced depression remains to be elucidated.

Functional magnetic resonance imaging improves our understanding of the neuroimaging mechanisms on SD and depression. As a voxel-wise, data-driven method, functional connectivity density mapping can analyze whole-brain functional communication and density distribution (17, 18); higher functional connectivity density (FCD) values represent those particular voxels that are more tightly connected than other voxels in functional connection. Acute total SD decreases brain activation in the default mode network (DMN), front-parietal attention network, and salience network (19–21). Perfusion magnetic resonance imaging (MRI) study has shown that regional cerebral blood flow changes in shift work were significantly correlated with depression (22). In addition, patients with depression were supposed to have lower levels of monoamine neurotransmitters, including serotonin (5-HT), norepinephrine (NE), and dopamine (23, 24). Altered 5-HT and NE levels were associated with depression both in the central and peripheral regions (25). However, few studies have focused on functional neuroimaging and monoamine neurotransmitters of SD after night shift work in ICU medical staffs.

The main purpose of this study was to display the general impact of SD in ICU medical staffs after night shift work on FCD, Hamilton Depression Rating Scale (HAMD) scores, and serum levels of 5-HT and NE. We hypothesized that (1) FCD changes in depression-related regions would be observed in ICU medical staffs after SD; (2) altered HAMD scores and the plasma levels of 5-HT and NE within participants were in line with depressive tendency; (3) ICU medical staffs tend to have higher depressive propensity after one night of shift work than when rested.

## Methods

### Participants

Twenty-two right-handed surgical ICU or anesthesia ICU medical staffs were initially recruited from The First Affiliated Hospital of Zhengzhou University, Henan, China. The study was conducted in line with the Declaration of Helsinki Declaration and approved by the Ethics Committee of Scientific Research and Clinical Trial of The First Affiliated Hospital of Zhengzhou University. All subjects received written informed consent and met the following inclusion criteria:

1. aged ≥ 18 years;
2. with regular shift work for more than 2 years (22);
3. 17-item HAMD score ≤ 20 at rest.

The exclusion criteria are given as follows:

1. intracranial space-occupying lesions;
2. pregnancy or lactation;
3. claustrophobia or history of psychiatric disorders.

Among them, one participant was excluded due to intracranial cavernous hemangioma. Finally, 21 subjects were included in this study.

### Depression Assessments

Depression was assessed using the HAMD score, which was widely used in patients with anxiety and depressive disorders (26). An experienced neurological physician assessed all subjects in the local hospital between 7 and 8 am. The HAMD scores were acquired twice on two occasions: one at rested wakefulness (RW) and the other after SD with one night of shift work. The two states were separated by at least 5 days. The questionnaires involved were anonymous.

### Enzyme-Linked Immunosorbent Assay

Blood samples were collected both at RW and after SD, which were performed after the HAMD assessment and MRI scan. Because the procedure of blood sample collection was invasive, only 17 paired samples were finally obtained. Blood was collected into ethylenediaminetetraacetic acid tubes (5 ml, for measurement of 5-HT and NE). Samples were left standing at room temperature for at least 30 min. The supernatant serum was removed after centrifuging at 3,000 × *g* for 10 min in a 4°C centrifuge. Then, a 600-μl liquid component was immediately transferred in Eppendorf tubes, which were stored at a −80°C refrigerator until further use. Enzyme-linked immunosorbent assay was performed to measure the concentrations of 5-HT (Elabscience, E-EL-0033c, China) and NE (Elabscience, E-EL-0047c, China) following the manufacturer's instruction (27).

### Magnetic Resonance Imaging Data Acquisition

The functional MRI (fMRI) data were acquired on the same 3.0 T Siemens MAGNETOM Trio scanner (Siemens, Erlangen, Germany) with a standard eight-channel head coil in the First Affiliated Hospital of Zhengzhou University, China. After resting for 30 min, all participants were advised to maintain the head in neutral and were made to relax with their eyes closed, stay awake, and keep nothing in mind during the MRI acquisition. Earplugs and pillowy foam pads were used throughout the scan. The imaging parameters of the echo-planar imaging sequence were as follows: repetition time/echo time = 2,000/30 ms, flip angle = 90°, field of view = 240 × 240 mm, voxel size = 3.4 × 3.4 ×4 mm3, matrix size = 64 × 64, and slice thickness/gap = 4.0/1 mm. There were 32 transverse slices, and the scanning time was 8 min. For this sequence, 240 volumes were collected.

High-resolution T1-weighted three-dimensional structural information for anatomical localization was acquired using a high-resolution sequence [repetition time = 2,000 ms, echo time = 30 ms, voxel size = 0.5 × 0.5 × 1 mm3; field of view = 256 × 256 mm; flip angle = 7^°^; and slice thickness = 1 mm (no gap)] to generate 176 slices.

### Functional Magnetic Resonance Imaging Data Preprocessing

Preprocessing of fMRI data was conducted using Data Processing Assistant for resting-state fMRI package (http://www.restfmri.net). The detailed procedure is as follows. The first 10 volumes of each participant were discarded to reduce the magnetization equilibrium until they could adapt to the noisy scanning surroundings. Slice timing was corrected among the remaining 230 volumes. Images were realigned with the threshold of no translational or rotational motion exceeding 2 mm or 2^°^. Frame-wise displacement was also calculated, which reflects the volume-to-volume mismatch in head position (28). The realigned functional images were spatially normalized to the standard Montreal Neurological Institute echo-planar imaging template and resampled to 3 × 3 × 3 mm3 voxels. Then, a 6-mm full width at half maximum Gaussian kernel was used to smooth the normalized images. Detrending was performed to reduce low-frequency linear drift. Several nuisance signals were regressed through a linear regression analysis, including the Friston 24-parameter model (29), white matter, and cerebral spinal fluid signal. To decrease the effects of low-frequency drifts and high-frequency noise, a band-pass filter (0.01–0.08 Hz) was performed on the time series of each voxel. Finally, to remove the effect of head motion and ensure the contiguous time points, scrubbing with cubic spline interpolation was used.

### Functional Connectivity Density Calculation

The individual FCD map was restricted to voxels in the gray matter mask and calculated on the basis of the method proposed by Tomasi and Volkow (17). For FCD maps, local FCD, long-range FCD, and global FCD (gFCD) maps were calculated. The correlation threshold was determined by the significance of a single functional connection (*P*-value). A functional connection (correlation coefficient) was considered significant if its *P* < 0.05 (Bonferroni corrected). Region of interest (ROI)-based FC analysis was performed on the ROIs that showed significant differences within subjects. The ROIs were defined as 6-mm radius spheres with the software SPM12 for FC analysis. The calculation of every last voxel constitutes the global FCD, which signifies the FCD distribution of the whole brain. The strength of the long-range FCD (lrFCD) was defined as the gFCD minus the local FCD (30). The obtained FCD maps were transformed to z-scores by subtracting the mean value and dividing by the standard deviation across gray matter voxels. We investigated the Spearman correlation of HAMD scores and concentrations of neurotransmitters after SD with encephalic regions showing significant differences in FCD as well.

### Statistical Analysis

Statistical analyses were conducted using IBM SPSS software (Chicago, IL, USA, version 25.0). Descriptive analysis was used to describe the baseline information. The measurement values were presented as mean ± standard deviation or the median (interquartile range) depending on its distribution. The Kolmogorov–Smirnov test was used for normally distributed data. A voxel-wise paired two-sample *t*-test was performed on the normalized FCD data to test the group differences between RW and SD sessions with age, sex, and education level as covariates. Comparison between HAMD scores and concentrations of 5-HT and NE within-subjects of the two time points were performed by paired two-sample *t*-tests in the presence of homogeneous variance, whereas in the case of heterogeneous variance, the Wilcoxon rank-sum test was utilized. Pairwise comparison of Spearman correlation coefficients was calculated between the FCD values with HAMD scores and the concentration of 5-HT and NE after SD. *P* < 0.05 was considered statistically significant.

## Results

### Demographic Characteristics

The characteristics of the study population are displayed in Table 1; all participants (15 women and 6 men) were aged between 24 and 38 years.

**Table 1:** Demographic and clinical characteristics of all participants.

**Characteristic**	**Participants**
Number of subjects	21
Age (years) (mean ± SD)	28.9 ± 3.4
Sex (female/male), *n*	15 / 6
Education (years) (mean ± SD)	16.2 ± 2.5
Department (surgical/anesthesia), *n*	9/12
Work experience (years) (mean ± SD)	6 ± 2.3

*SD, standard deviation*.

### Hamilton Depression Rating Scale Scores and Peripheral Serum Concentration of Serotonin and Norepinephrine

**Figure 1:**
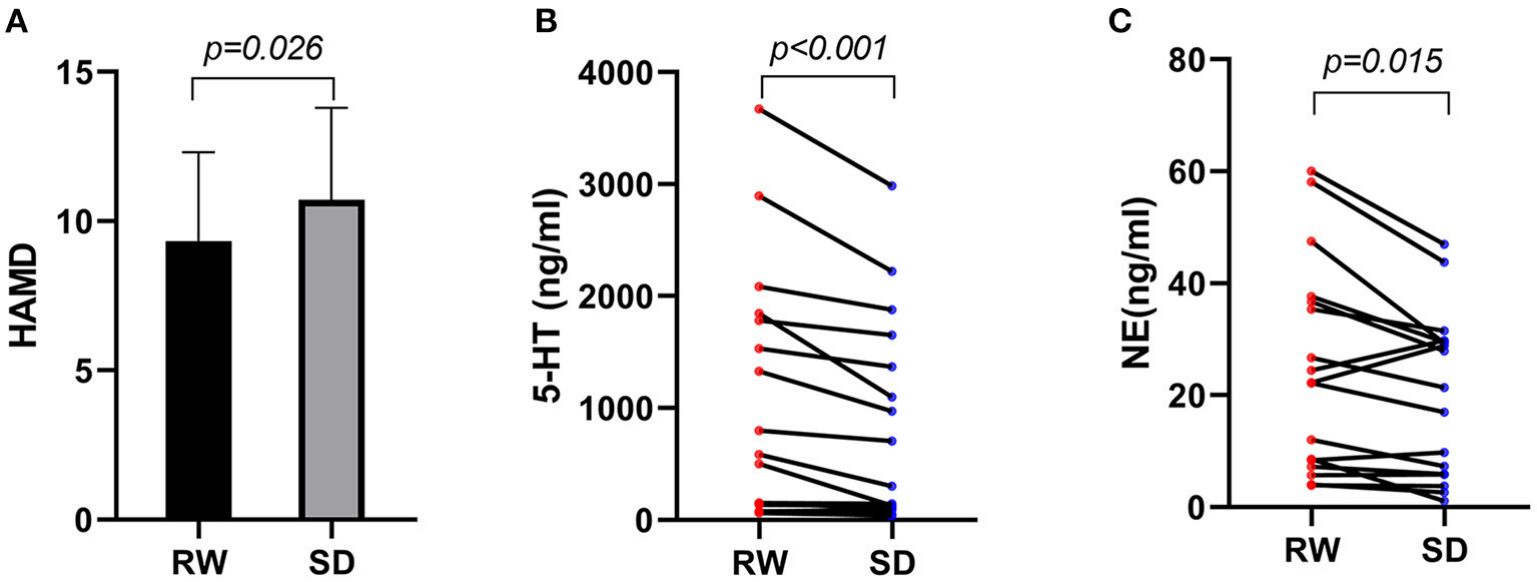
**(A)** HAMD scores within subjects at RW and after SD, *n* = 21. **(B)** 5-HT serum concentration of participants at RW and after SD, *n* = 17. **(C)** NE serum concentration of participants at RW and after SD, *n* = 17. HAMD, Hamilton Depression Rating Scale; RW, rested wakefulness; SD, sleep deprivation; 5-HT, serotonin; NE, norepinephrine.

For all the subjects, the HAMD scores ranged from 5 to 16. Paired *t*-test manifested that the HAMD scores of participants (Figure 1A) after SD (10.7 ± 3.1) were significantly higher (*P* < 0.05) than RW (9.3 ± 2.9). As presented in Figure 1B, Wilcoxon rank-sum test showed that the serum concentration of 5-HT in participants after SD [301.4 (106.1, 1,508.6)] was meaningfully decreased compared with that at RW [583.9 (140.8, 1,814.4)] (*P* < 0.001). Besides, the NE concentration of participants (Figure 1C) after SD [21.4 (5.9, 29.6)] was notably lower than that at RW [22.3 (7.8, 37.2)] (*P* < 0.05).

### Within Subjects Differences in Functional Connectivity Density

**Figure 2:**
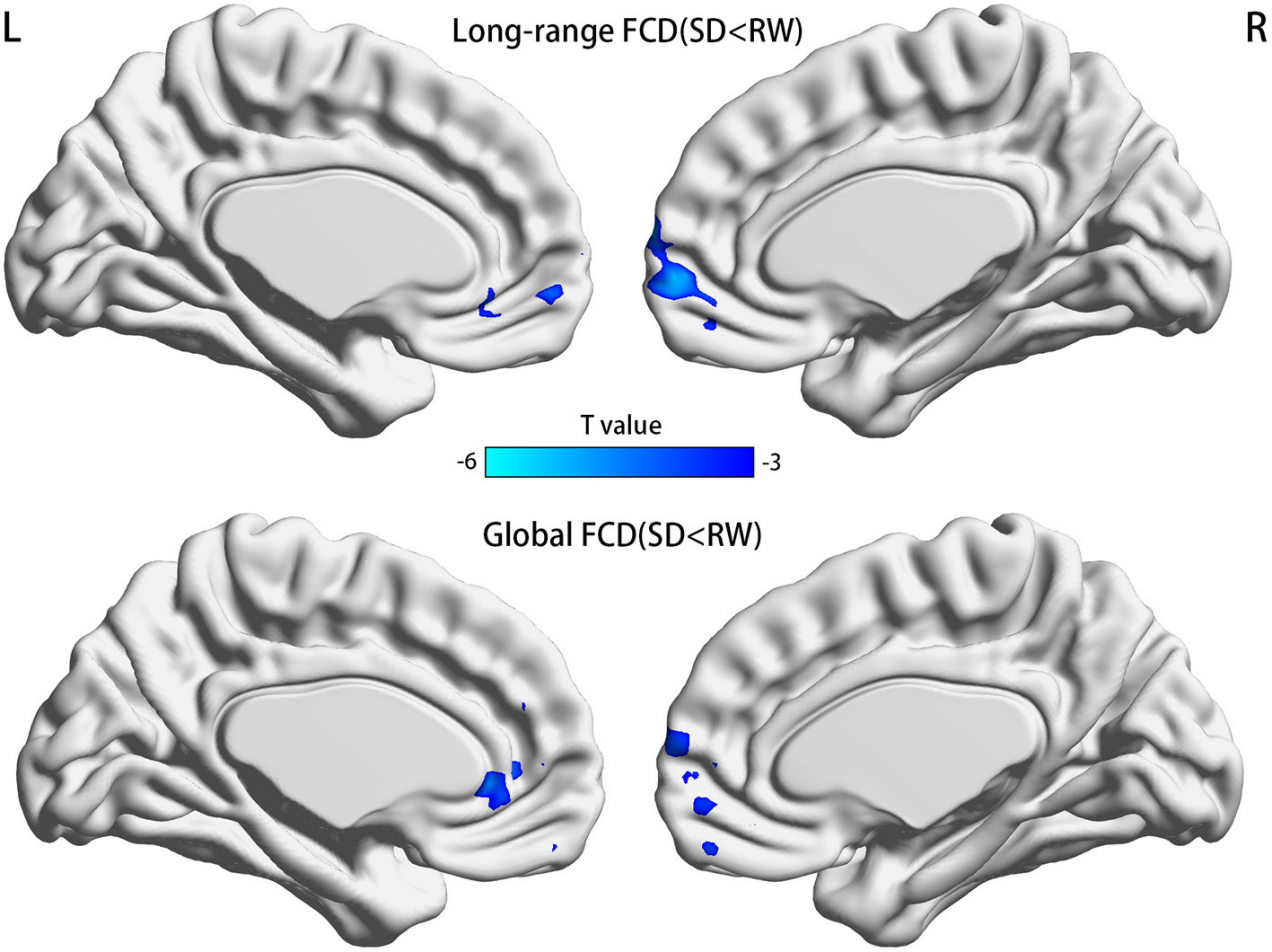
Significant differences of FCDs between RW and SD, *n* = 21. Significantly decreased regions were shown with a negative *t*-value. FCD, functional connectivity density; RW, rested wakefulness; SD, sleep deprivation; R, right; L, left.

FCDs within subjects between RW and SD are shown in Figure 2 and Table 2. Compared with RW, participants after SD had significantly decreased FCDs in the medial frontal gyrus of the right frontal lobe and the anterior cingulate gyrus of the left limbic lobe. Most of these FCD hubs belonged to the DMN and had an intersection with the medial prefrontal cortex (mPFC). Specifically, the significantly decreased gFCD was located in the right medial frontal gyrus and anterior cingulate gyrus. Meanwhile, the significantly decreased lrFCD was mainly found in the medial frontal gyrus. Statistical maps were corrected with the Gaussian random field method at the threshold of voxel *P* < 0.001, cluster *P* < 0.05 (31).

**Table 2:** Significant differences of FCDs within subjects between RW and SD.

**Parameter (SD < RW)**	**Brain regions**	**MNI coordinates**	**Cluster size**	**Peak *t* value**
		**X Y Z**		
lrFCD	Medial frontal gyrus	6 57 0	230	−5.40
gFCD	Medial frontal gyrus/Anterior cingulate gyrus	3 57 3	321	−5.78

*MNI, Montreal Neurological Institute*.

### Correlation Analysis

**Figure 3:**
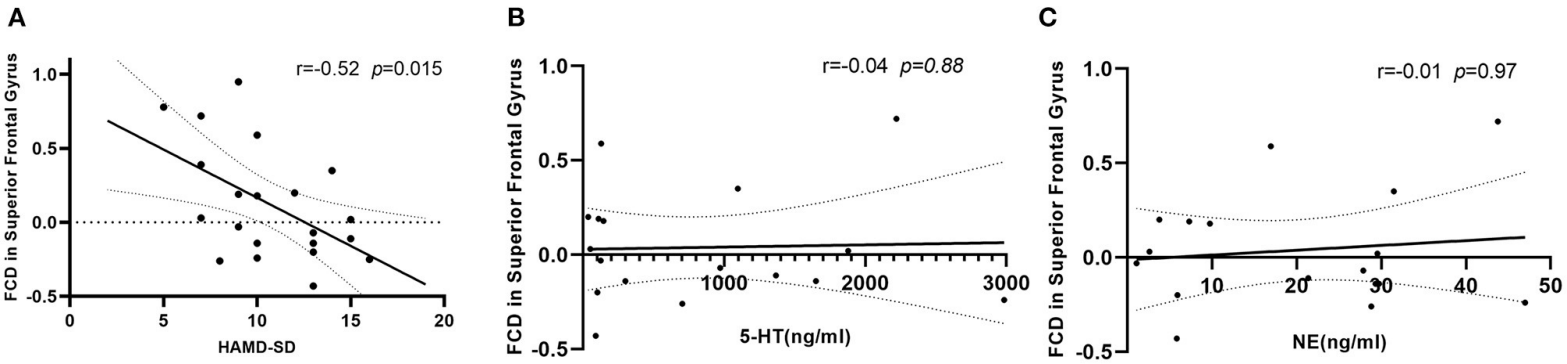
**(A)** Correlation between value of lrFCD in right medial frontal gyrus and HAMD scores after SD, *n* = 21. **(B)** Correlation between value of lrFCD in right medial frontal gyrus and concentration of 5-HT after SD, *n* = 17. **(C)** Correlation between value of lrFCD in right medial frontal gyrus and concentration of NE after SD, *n* = 17. FCD, functional connectivity density; HAMD, Hamilton Depression Rating Scale; SD, sleep deprivation; 5-HT, serotonin; NE, norepinephrine.

The analysis of Spearman rank correlation showed a significant negative correlation between the value of lrFCD in the right medial frontal gyrus and the HAMD scores after SD (*r* = −0.52, *p* = 0.015) (Figure 3A). However, there was no significant correlation between FCDs and the concentration of 5-HT and NE after SD (Figures 3B,C).

## Discussion

The present study demonstrates that acute SD after night shift work in ICU medical staffs induced deactivation in part of brain areas related to DMN. The specific manifestations included decreased gFCD in the right medial frontal gyrus and the anterior cingulate gyrus and decreased lrFCD in the medial frontal gyrus. These brain areas were known as the core components of the DMN (32). Furthermore, lrFCD alterations in those relevant brain regions were negatively correlated with HAMD scores after SD. In addition, serum levels of 5-HT and NE in participants after SD decreased significantly compared with those at RW. Although all the medical staffs were not able to meet the diagnostic criteria of depression, an increased risk of a depressive tendency among them was observed in our study. Medical staffs' own health could be disturbed by shift work; this phenomenon is general and deserves more attention.

Shift work is usually considered as an increasingly prevalent schedule that includes working other than the traditional period of 8:00 am to 6:00 pm (33). Night shift work requires individuals to be prevented from sleeping, thus disrupting normal circadian rhythms (34). A permanent night shift may lead to shift-work disorder (35). ICU is the setting for providing continuous medical services with long working hours and shift cycles. Rotating night shift work and intensive work are common in ICU medical staffs. Based on this phenomenon, night-shift-related SD is usually accompanied by ICU medical staffs. Thus, SD turns into night shift work-related consequences (12). A recent study conducted in Australia shows that night shift work can aggravate fatigue, sleepiness, and psychomotor vigilance in ICU physicians (36). Only one-night SD can disturb molecular clearance ability of certain brain regions, which was not able to be totally compensated even after 48 h of sufficient sleep (37). Moreover, SD can weaken the cognitive performance; then, after 1–2 nights of recovery, the competence returns to baseline (38, 39).

Heavy workload and high frequency of shift work are common in ICU medical staffs. Importantly, exposure to stress such as SD can result in structural and functional alterations of multiple human brain regions (40, 41). In particular, the mPFC, as well as the anterior cingulate gyrus, has been thought to be associated with emotional processing (42). The damage of mPFC may result in the impairment of cognitive process, motivation, and regulation of emotion (43, 44), which plays an important role in major depression (45). DMN is an integral part of the resting brain network, which consists of two parts: the anterior DMN and the posterior DMN. Relevant brain regions encompass the medial prefrontal lobe, precuneus/posterior cingulate, inferior parietal lobule, and lateral temporal cortex (46, 47). DMN is the most active network at rest, whereas turned into deactivated during task (48). When performing the assignment, subjects' task-related brain regions would be activated; simultaneously, the DMN activation was inhibited (49). Maintenance of this antagonistic relationship in individuals plays a key role in accomplishing the task. Meanwhile, DMN has a critical role in internally generated tasks such as cognitive control and emotional appraisal (50, 51). Previous studies found that participants' DMN connectivity altered after being exposed to SD and revealed that SD was associated with lower DMN functional connectivity (52). The abnormal function of DMN and its interactions with other networks were found to play an important role in depression (53). Our result that decreased lrFCD in the medial frontal gyrus among participants after shift work was in line with this finding. In another study, it has been shown that SD could significantly reduce brain activation in multiple regions compared with RW, including bilateral intraparietal sulcus, bilateral insula, right prefrontal cortex, medial frontal cortex, and right parahippocampal gyrus (19). However, our study showed that SD only affected the medial frontal gyrus and anterior cingulate cortex. This discrepancy might be explained by the reason that we included different participants. ICU medical staffs need to maintain high strength of clinical work and take care of seriously ill patients all through the night, whereas participants from the previous study were allowed to listen to music, read books, or play computer games (54).

The monoamine deficiency hypothesis (55) and the hypothalamic–pituitary axis (56) dysregulation have been speculated to clarify the underlying biological basis of depression. The monoamine deficiency hypothesis explores the association between depleted levels of monoamine neurotransmitters in the central nervous system with depression. Depression is usually caused by reduced neurotransmitters in the brain, and increasing these substances could have an antidepressant effect (57). Thus, the 5-HT and NE reuptake inhibitors and selective 5-HT reuptake inhibitors are considered to be the first choice of treatment for depression (58). Although the pathophysiology of depression is still unknown, there is significant evidence for the abnormalities of the 5-HT and NE systems in depressive disorders (23). Even if the diagnosis of depression does not depend on specific levels of monoamine neurotransmitters, it seems more important to understand their variation tendency. The results of our study demonstrated that serum concentrations of 5-HT and NE decreased significantly after SD. In the present study, we merely measured serum monoamine levels instead of cerebrospinal fluid (CSF). However, fluctuations in the levels of monoamine neurotransmitters and their metabolites can differ between peripheral blood and CSF (59). This phenomenon is a likely explanation for the absence of a statistically significant correlation between serum neurotransmitters with FCDs.

Some limitations should be noted in this study. First, we did not analyze work intensity and sleep quality in our study, which might affect the result of our study. Second, we did not measure the levels of monoamine neurotransmitters in CSF. Third, we included a relatively small sample size and more female staffs, which may affect the interpretation of the results. Thus, future studies with a larger sample size are needed to address these important issues.

## Conclusion

In conclusion, our study suggested that SD after night shift work can induce depressive tendency in ICU medical staffs, which might be related to disturbed function of mPFC and altered serum levels of monoamine neurotransmitters.

## Data Availability Statement

The raw data supporting the conclusions of this article will be made available by the authors, without undue reservation.

## Ethics Statement

The studies involving human participants were reviewed and approved by the Ethics Committee of Scientific Research and Clinical Trial of the First Affiliated Hospital of Zhengzhou University. The patients/participants provided their written informed consent to participate in this study.

## Author Contributions

HY and MJ conceived and designed the study. MJ and J-JY supervised the conduct of the study. HY, ChW, LC, and YaL are responsible for data acquisition. HY, YC, and CoW analyzed the data and took responsibility for the paper. YiL, LC, and MJ assisted with the literature review. HY and ChW drafted the initial manuscript. J-JY and MJ reviewed and revised the manuscript. All authors read and approved the final manuscript.

## Funding

This research study was supported by the National Natural Science Foundation of China (Grant No. 8197051831) and the Zhongyuan Science and Technology Innovation Leadership Program of Henan Province, China (Grant No. 2060299).

## Conflict of Interest

The authors declare that the research was conducted in the absence of any commercial or financial relationships that could be construed as a potential conflict of interest. The reviewer CQL declared a shared affiliation, with no collaboration, with the authors YiL at the time of the review.

## Publisher's Note

All claims expressed in this article are solely those of the authors and do not necessarily represent those of their affiliated organizations, or those of the publisher, the editors and the reviewers. Any product that may be evaluated in this article, or claim that may be made by its manufacturer, is not guaranteed or endorsed by the publisher.
